# The risk of menstrual abnormalities after tubal sterilization: a case control study

**DOI:** 10.1186/1472-6874-5-5

**Published:** 2005-05-02

**Authors:** Mehri Jafari shobeiri, Simin AtashKhoii

**Affiliations:** 1 Department of Obstetrics and Gynecology, Al-zahra Hospital, Tabriz University of Medical Sciences, South Artesh Ave., Tabriz, Iran; 2 Department of Anesthesia, Al-zahra Hospital, Tabriz University of Medical Sciences, South Artesh Ave., Tabriz, Iran

## Abstract

**Background:**

Tubal sterilization is the method of family planning most commonly used. The existence of the post-tubal-ligation syndrome of menstrual abnormalities has been the subject of debate for decades.

**Methods:**

In a cross-sectional study, 112 women with the history of Pomeroy type of tubal ligation achieved by minilaparatomy as the case group and 288 women with no previous tubal ligation as the control group were assessed for menstrual abnormalities.

**Results:**

Menstrual abnormalities were not significantly different between the case and control groups (p = 0.824). The abnormal uterine bleeding frequency differences in two different age groups (30–39 and 40–45 years old) were statistically significant (p = 0.0176).

**Conclusion:**

Tubal sterilization does not cause menstrual irregularities.

## Background

Tubal sterilization is the most commonly used method of family planning. In 1990 the corresponding percentage of married women in reproductive age who used sterilization was 22% in developing countries and the corresponding percentage in developed countries was 11%. These women represented 44% and 18% of all contraceptive users in developing and developed countries, respectively. Questions regarding the existence of a post tubal ligation syndrome of menstrual abnormalities continue. Questions arose initially when Williams and colleagues reported in 1951 that sterilized women had a higher than expected occurrence of menorrhagia and metrorrhagia [1]. After that the existence of a post-tubal-ligation syndrome of menstrual abnormalities has been debated for decades [2]. Many authors have investigated the sequelae of female sterilization [2-9]. Increased premenstrual distress, heavier and more prolonged menstrual bleeding, and increased dysmenorrhea have been reported [3]. However, failure to control for use of oral contraceptives, age, obesity, parity, interval since sterilization, or type of sterilization may have effects on the results of these studies [1,3]. Because of the importance of this debate, we compared the occurrence of menstrual abnormalities in women with and without a prior history of tubal ligation.

## Methods

This cross sectional case control study has been carried out on 500 women at Al-zahra hospital during 1999 to 2001 to assess the effect of tubal sterilization on the menstrual cycle. 260 women with abnormal uterine bleeding referred for diagnostic curettage, and 240 healthy women under the coverage of the hospital family planning center were selected randomly, and all were assessed for tubal ligation.

All women aged 30 to 46 were selected from a low-income urban population, with body weight between 50 to 90 kg. In the abnormal uterine bleeding group, those who had intrauterine device (IUD), leiomyoma on sonography, uterine size of greater than 9 cm or suffered from medical disorders were excluded from the study. Of 260 patients with menstrual irregularities, 30 subjects were excluded from the study. From the remaining 230 subjects, assessed for tubal sterilization, 87 patients had tubal ligation. Of 240 healthy women assessed for tubal ligation, 95 had previous tubal ligation. Totally 182 subjects with previous tubal ligation (case) and 288 subjects with no history of previous tubal ligation (control) were compared for abnormal uterine bleeding. Those subjects in the case group who had menstrual abnormalities, IUD, medical disorders or were on hormonal contraception, during the first year prior to the sterilization were excluded from the study. Those who were at least 30 and at most 40 years of age by the time of tubal ligation and had Pomeroy type of interval tubal ligation via minilaparatomy were included the study. Finally, considering the exclusion and inclusion criterias, 112 subjects remained in the case group and 288 with no tubal ligation in the control group were evaluated for menstrual abnormalities. Information on demographic, obstetrics, medical and menstrual bleeding pattern of all subjects were obtained. Women were asked about the duration and amount of bleeding, and length of cycle (number of days from the beginning of one menstrual period to the beginning of the next one). A menstrual interval of 21 to 35 days was considered normal. A menstrual interval shorter than 21 days was defined as polymenorrhea. Duration of flow of 7 days or less was considered normal. A patient's self-described history of normal or heavy blood loss was indicative of the amount of flow. Regularly timed heavy bleeding and duration of flow greater than 7 days were considered menorrhagia and hypermenorrhea respectively. Excessive and prolonged bleeding that occurred irregularly was defined as menometrorrhagia.

Data was analyzed by the SPSS statistical software (version, 12) and compared with the chi-square test. P values of 0.05 or less were considered as statistically significant.

## Results

By considering the exclusion and inclusion criterias, 112 tubal ligated (case) and 288 non-tubal ligated subjects (control) were evaluated for menstrual abnormalities. Of 112 subjects in the case group, 57 (50.8%) had menstrual abnormalities. The corresponding figure in the control group was 143, accounting for 49.6% of the studied subjects in this group. The results of chi-square analysis, indicate that there was no significant difference in the menstrual abnormalities between two groups, χ^2 ^= 0.050, p = 0.824.

The highest frequency of the menstrual abnormalities in the case group was 54.3% for the group aged between 30–39 while in the control group this value was 65% for those aged 40–45. There was significant difference in the menstrual abnormalities frequency of two groups by different age groups, χ^2 ^= 9.06, p = 0.0176 (Table 1).

**Table 1:** Demographic and obstetrical information of subjects with menstrual irregularities in case and control groups

	**(57) case group**	**(143) control group**	**df**	**pv**
				
	**No. (%)**	**No. (%)**		
**Age groups(year):**				
30–39	31(54.3)	50(34.96)	1	0.0176819
40–45	26(45.6)	93(65.1)		
**Parity groups (No.):**				
2–4	8(14)	31(22)	2	0.3819013
5–7	28(50)	58(40)		
>7	21(36)	54(38)		

**Figure 1:**
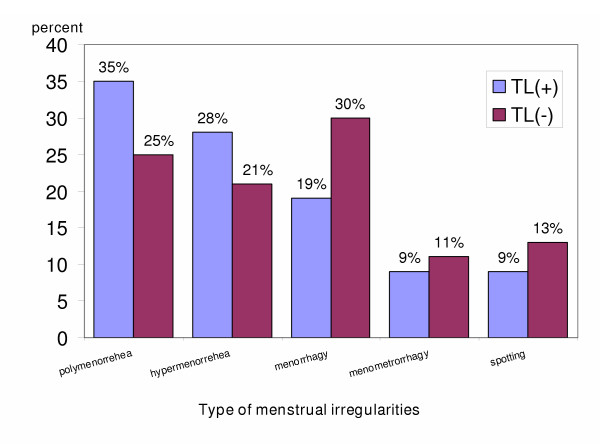
Comparison of the menstrual irregularities type of case and control groups

Type of abnormal bleeding is given in Figure 1. The most common type of menstrual changes in case and control groups was polymenorrhea (35%) and menorrhagy (30%) respectively. The differences were not significant, χ^2 ^= 6.93 p = 0.2260.

The frequency onset of abnormal bleeding after sterilization in the case group was 61% during the first year, 34% in 2–5 years after sterilization and 5% over 5 years.

The menstrual abnormality frequency distributions by different parity groups in the case and control groups are shown in Table 1. The most common menstrual abnormalities frequency which belonged to parity of 5–7, was 50% and 40% in case and control groups respectively. The parity differences between two groups were not significant, χ^2 ^= 1.93 p = 0.3819.

The most common histologic findings in case and control groups were proliferative endometrium 31.6% and anovulatory cycle (28.7%) respectively. There was no significant difference in the histologic finding of two groups, χ^2 ^= 5.351, p = 0.253.

## Discussion

There are some factors other than sterilization *per se *that may have influences on post sterilization menstrual changes. Two such factors are the use of oral contraceptives and IUD. The women who use oral contraceptive may have some menstrual changes after sterilization attributable solely to cessation of oral contraceptive use. In order to exclude the interventional effect of IUD and oral contraceptive, we included patients who did not use them during one year before sterilization. Since the type of tubal sterilization may have effects on study results, we included only Pomeroy type of interval sterilization by minilaparatomy. The results are similar to those of Gentile et al[3]., Bernard et al[4], Peterson et al[2], who showed no significant changes in menstrual cycle characteristics in women with or without tubal ligation. Concerning the demographic information including the socioeconomic status among the case and control groups, all participants were of a low-income population. In the unadjusted analysis, when the sterilized groups were compared to the control group, slight but not statistically significant changes were noted in menstrual indices. The results are similar to those of Peterson et al[2], Bhiwandiwala et al[11], who showed no menstrual pattern changes following sterilization.

Although we had excluded patients who were on hormonal contraceptives and had IUD, we found that most of the menstrual changes occurred at first year of sterilization (61%). After first year of sterilization the menstrual changes decreased to 34% in 2–4 years and 5% after 5 or more years of sterilization. The results are similar to those of Parsanezhad et al[12], who found that almost all menstrual changes occurred between 6 and 24 months after sterilization. Thus it may be concluded that sterilization related menstrual changes during the first years of sterilization may occur due to some psychological reaction to tubal ligation. DeStefano et al[13], in their long term follow up of sterilized women found an increased risk of menstrual abnormalities even after a long period of 49 to 87 months after sterilization. These late menstrual changes are difficult to explain, because it is not easy to postulate a physiologic mechanism that would take more than 4 years to develop and adversely affect menstrual cycles. Our results are dissimilar to those of Kasonde and Bonnar[14] who did not find any increased menstrual blood loss up to 6–12 months after sterilization. It seems that different results of these studies may because Kasonde and Bonnar objectively measured blood loss whereas this study relied on subjective self reported amounts of blood loss.

Shy et al[15] believe that menstrual changes effect of sterilization depends on age at the time of sterilization. Women who undergo sterilization between 20 and 29 years of age have more menstrual irregularities than women who undergo the procedure after age 30. In order to exclude this factor, we included only the patients with at least 30 and at most 40 years of age by the time of sterilization. The results show that the most common age group of menstrual irregularities is 30–39 years (Table 1). These results are similar to those of Wilcox et al[16] and Shy et al[15] who found that sterilization at younger ages leads to more menstrual irregularities than sterilization at older ages.

## Conclusion

Women who have undergone a Pomeroy type of tubal ligation have no more menstrual abnormalities than those without tubal ligation. Sterilization at younger ages has more affect on menstrual irregularities than sterilization at older ages.

It seems that more frequency of menstrual changes at first year of sterilization is due to other factors such as psychiatric problems. Further studies on psychiatric changes of sterilization are mandatory to evaluate its effects on immediate post sterilization menstrual irregularities.

## Competing interests

The author(s) declare that they have no competing interests.

## Authors' contributions

MJSH is the principle investigator and was involved in planning, coordinating the research, sample collection, handling, writing and editing of the manuscript, SA was involved in coordinating and supervising data entry and analysis and was involved in planning, coordination of the research at the Hospital family planning center, and reviewing of the paper. Both authors read and approved the final manuscript.

## Pre-publication history

The pre-publication history for this paper can be accessed here:


